# A role for APP in Wnt signalling links synapse loss with β-amyloid production

**DOI:** 10.1038/s41398-018-0231-6

**Published:** 2018-09-20

**Authors:** Christina Elliott, Ana I. Rojo, Elena Ribe, Martin Broadstock, Weiming Xia, Peter Morin, Mikhail Semenov, George Baillie, Antonio Cuadrado, Raya Al-Shawi, Clive G. Ballard, Paul Simons, Richard Killick

**Affiliations:** 1 Maurice Wohl Clinical Neuroscience Institute, King’s College London, 5 Cutcombe Road, London, SE5 9RT UK; 2 Centro de Investigación Biomédica en Red sobre Enfermedades Neurodegenerativas (CIBERNED), Instituto de Investigación Sanitaria La Paz (IdiPaz), Autonomous University of Madrid (UAM), Madrid, Spain; 3 Department of Psychiatry, Warneford Hospital, University of Oxford, Oxford, OX3 7JX UK; 4 Boston University School of Medicine, Boston, MA USA; 5 Department of Veterans Affairs, Geriatric Research Education and Clinical Center, Bedford, MA USA; 6 Institute of Cardiovascular and Medical Science, University of Glasgow, Glasgow, G12 8QQ Scotland; 7 Wolfson Drug Discovery Unit, Centre for Amyloidosis and Acute Phase Proteins, Royal Free Campus, University College London, Rowland Hill Street, London, NW3 2PF UK; 8 University of Exeter Medical School, Exeter, UK

## Abstract

In Alzheimer’s disease (AD), the canonical Wnt inhibitor Dickkopf-1 (Dkk1) is induced by β-amyloid (Aβ) and shifts the balance from canonical towards non-canonical Wnt signalling. Canonical (Wnt-β-catenin) signalling promotes synapse stability, while non-canonical (Wnt-PCP) signalling favours synapse retraction; thus Aβ-driven synapse loss is mediated by Dkk1. Here we show that the Amyloid Precursor Protein (APP) co-activates both arms of Wnt signalling through physical interactions with Wnt co-receptors LRP6 and Vangl2, to bi-directionally modulate synapse stability. Furthermore, activation of non-canonical Wnt signalling enhances Aβ production, while activation of canonical signalling suppresses Aβ production. Together, these findings identify a pathogenic-positive feedback loop in which Aβ induces Dkk1 expression, thereby activating non-canonical Wnt signalling to promote synapse loss and drive further Aβ production. The Swedish familial AD variant of APP (APP_Swe_) more readily co-activates non-canonical, at the expense of canonical Wnt activity, indicating that its pathogenicity likely involves direct effects on synapses, in addition to increased Aβ production. Finally, we report that pharmacological inhibition of the Aβ-Dkk1-Aβ positive feedback loop with the drug fasudil can restore the balance between Wnt pathways, prevent dendritic spine withdrawal in vitro, and reduce Aβ load in vivo in mice with advanced amyloid pathology. These results clarify a relationship between Aβ accumulation and synapse loss and provide direction for the development of potential disease-modifying treatments.

## Introduction

The early stages of Alzheimer’s disease (AD) are characterised by the progressive loss of synapses and increased levels of β-amyloid (Aβ), leading to cognitive impairment, dementia and a protracted death. As the precursor of the Aβ peptides^1^ and the first familial Alzheimer’s disease (FAD) gene to be identified^2^, the β-amyloid precursor protein (APP) holds a central position in AD neuropathology, while the synaptotoxic effects of soluble Aβ species are well documented^3–5^. However, the precise relationship between these two processes and how they contribute to the clinical disease are unclear. APP is a type 1 transmembrane protein and the generation of Aβ occurs in endosomes from APP molecules upon internalisation by clathrin-mediated endocytosis^6,7^. While the functions of APP are not fully understood, evidence for a role in cell signalling as one function has been accumulating^8,9^. As endocytosis and subsequent proteolytic cleavage is a fundamental mechanism for downregulating cell surface receptors, it is possible that the processing of APP and subsequent production of Aβ might be related to these signalling activities.

We^10^, and others^11^, have identified Dickkopf-1 (Dkk1) as a key player in AD. Increased expression of Dkk1 has been shown in post-mortem AD brain and in animal models of Aβ pathology^11^, and its expression is induced in neuronally enriched cultures as an early response to Aβ^10^. Dkk1 drives multiple aspects of Aβ-mediated neurotoxicity, including synapse loss through effects on Wnt signalling^12–14^. Dkk1 is known principally as an inhibitor of the Wnt-β-catenin, or canonical Wnt, pathway. However, we have demonstrated that concomitant with this antagonism Dkk1-induced activation of the non-canonical Wnt-planar cell polarity (Wnt-PCP) pathway is also required for Aβ-driven synapse disassembly to occur^14^. At synapses, the Wnt-β-catenin and Wnt-PCP Wnt pathways have opposing actions which, under normal physiological conditions, work in concert to maintain synaptic homeostasis^15–19^. Interestingly, recent evidence suggests that APP has a role in Wnt signalling^9^, raising the possibility of an intimate molecular link between synapse homeostasis and APP function. As such it is of great importance to elucidate the role of APP in Wnt signalling and its impact on synapse stability, and what effects there might be of Wnt signalling on APP processing and Aβ production.

Here we provide evidence that APP is a co-activator of both Wnt-β-catenin and Wnt-PCP signalling through specific interactions with the Wnt co-receptor proteins LRP6 (LDL-receptor related protein 6) and Vangl2 (van Gogh-like 2), respectively. Under conditions that promote Wnt β-catenin signalling, which enhances synapse stability, the Aβ production is reduced. Conversely, activation of Wnt-PCP, which drives synapse retraction, results in an increase in Aβ production. These data provide a mechanism that directly links Dkk1-induced synapse loss with raised Aβ production. As Dkk1 expression is induced by Aβ^10,11^, there exists a positive feedback loop in which Aβ enhances its own production by inducing Dkk1, which then promotes Wnt-PCP activity and further Aβ production: succinctly, Aβ synaptotoxicity drives Aβ production. Importantly, we demonstrate the in vitro effects of Aβ can be blocked by antagonising Wnt-PCP with the drug fasudil and, that when administered peripherally, the drug also dramatically lowers brain levels of both soluble and insoluble Aβ in vivo in an amyloid-based mouse model of AD.

## Results

### APP potentiates Wnt signalling

**Fig. 1:**
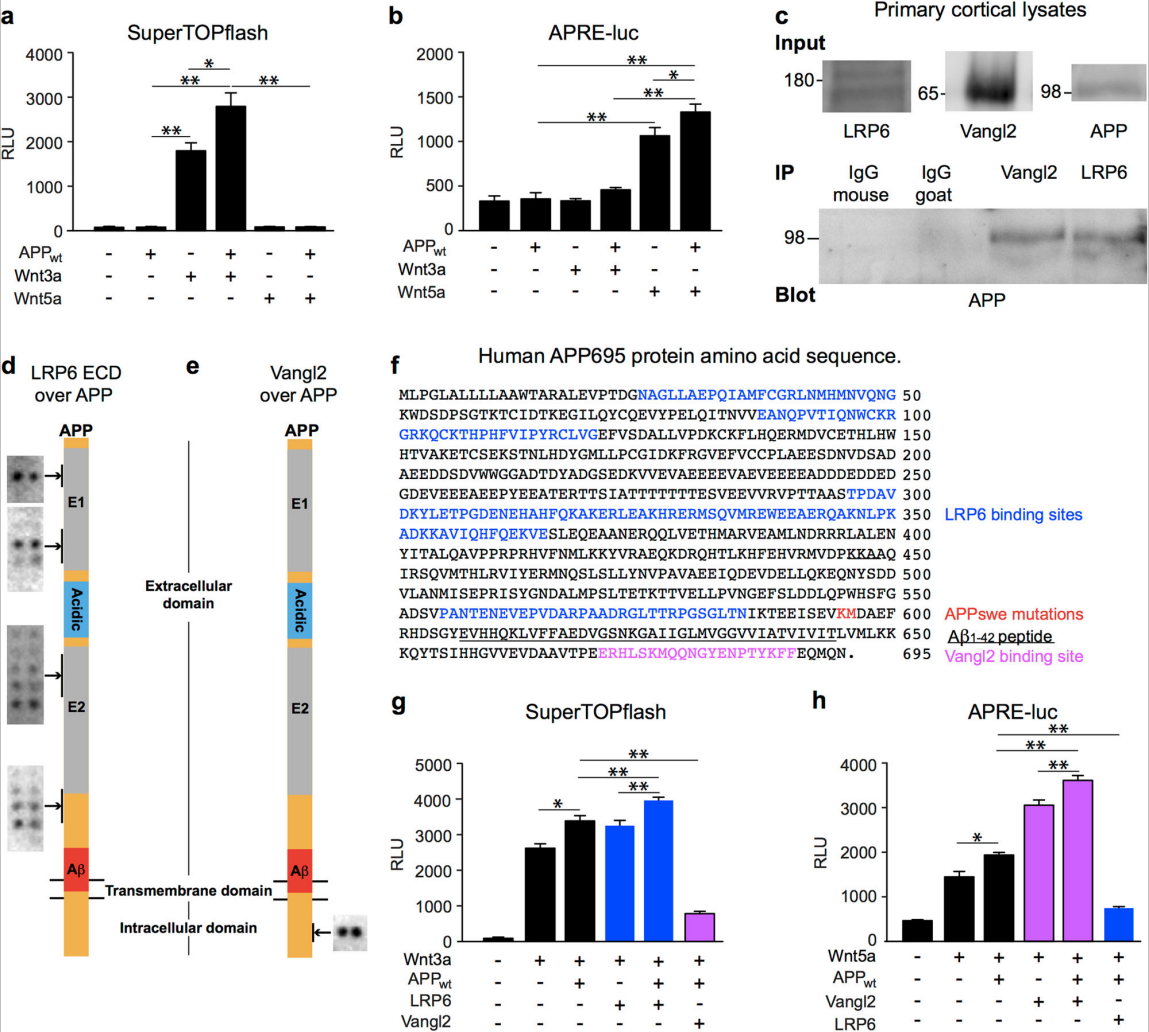
APP co-activates Wnt signalling and interacts with Wnt co-receptors LRP6 and Vangl2. **a** APP overexpression enhanced Wnt-β-catenin signalling as measured by TCF/LEF transcriptional activity using SuperTOPflash luciferase reporter assay in HEK293A cells; activity was measured in relative light units (RLU). The enhancement of Wnt-β-catenin signalling was only detectable in cells also overexpressing Wnt3a (a “canonical Wnt”) and not in the presence of the “non-canonical Wnt”, Wnt5a. **b** APP overexpression also enhanced Wnt-PCP signalling as measured by AP1 transcriptional activity (APRE-luciferase) in HEK239A cells. The enhancement of Wnt-PCP signalling was only detectable in cells also overexpressing Wnt5a and not in the presence of Wnt3a. **c** APP co-immunoprecipitated from lysates of primary rat cortical neurons with Wnt-β-catenin co-receptor protein, LRP6 and with Wnt-PCP co-receptor protein, Vangl2. (The full blots are presented in the supplemental data.) **d** Schematic illustrating the sites of APP-LRP6 interaction determined by peptide array hybridisation. Overlay of APP peptides with recombinant extracellular domain (ECD) of LRP6 demonstrated binding between the LRP6 and APP ECDs. **e** Schematic illustrating the site of APP-Vangl2 interaction as elucidated by peptide array. Overlay of APP peptides with HEK293A lysates overexpressing Vangl2-HA demonstrated binding of Vangl2 to a single site in the APP-ICD. **f** Schematic of the precise binding regions of LRP6/Vangl2 binding within the APP sequence. Binding of LRP6 is restricted to the ECD with one region lying close to the site affected by the Swedish APP mutation. In contrast, Vangl2 binding at the APP-ICD occurs at the YENPTY motif, which has been shown to control APP internalisation. **g** Overexpression of LRP6 enhanced the activity of APP in Wnt-β-catenin signalling as measured by TCF/LEF transcriptional activity using SuperTOPflash luciferase reporter assay in HEK293A cells. **h** Overexpression of Vangl2 enhanced the activity of APP in Wnt-PCP signalling as measured by APRE luciferase reporter assay in HEK293A cells. Data are plotted as mean+/− standard deviation (*n* ≥ 3). Statistical significance was determined by one-way ANOVA and post-hoc Tukey’s test (*p < 0.05; **p < 0.01); *p* values are indicated for key comparisons only

It has previously been reported that APP is a component of the Wnt-PCP molecular machinery^9^. To confirm this, and to investigate any possible role of APP in Wnt signalling, we used reporter gene assays in HEK293A cells, with stimulation by Wnt3a (a type-1 pro canonical Wnt) or by Wnt5a (a type-2 pro non-canonical Wnt) (Fig. 1a, b). Overexpression of APP alone did not markedly change the activity of either Wnt signalling pathway. APP did though potentiate both Wnt3a-driven Wnt-β-catenin signalling and Wnt5a-driven Wnt-PCP signalling. These findings confirm the previously reported involvement of APP in Wnt-PCP signalling, and identify a new role of APP in Wnt-β-catenin signalling. Expression of APP did not alter the specificity of pathway activation by Wnt3a and Wnt5a, which selectively activated Wnt-β-catenin and Wnt-PCP signalling, respectively.

### APP interactions with LRP6 or Vangl2 differentially modulate Wnt signalling

APP has previously been shown to interact with Vangl2^9^, a Wnt-PCP-specific co-receptor protein, and independently has been shown to bind LRP6^20^, a receptor component of the Wnt-β-catenin pathway^21^. However, it is unclear how these APP interactions impact Wnt-PCP or Wnt-β-catenin signalling activity. Given the importance of this for our understanding of AD pathology, we confirmed binding between endogenous APP and LRP6 or Vangl2 proteins in rat primary cortical neuronal cultures by co-immunoprecipitation (Fig. 1c), confirming the data previously obtained by exogenous overexpression of the proteins in HEK293 cells^9,20^. We then delineated the precise binding regions between APP and LRP6 or Vangl2 using an array of overlapping peptides spanning the full length of human APP_695_ [25mer peptides, each with a 5 aa shift] (Fig. 1d). We observed binding of the LRP6 extracellular domain at four sites in the extracellular domain of APP; two within the E1 domain and two in the E2 domain. One of these interactions maps to amino acids 555–584, very close to the β-secretase cleavage site, which lies between residues 596 and 597. These data are in agreement with the previous report that LRP6 and APP interact through their extracellular domains^20^.

Vangl2 binding was observed at a single site in the APP intracellular domain (Fig. 1e). Notably, the region of APP to which Vangl2 binds includes the YENPTY motif. This motif is has previously been identified^22^ as a binding site for several proteins known to interact with APP, and is also a signal for clathrin-mediated endocytosis^23^.

To determine the functional significance of these direct physical interactions of APP with LRP6 and Vangl2, we assessed the effects of LRP6 and Vangl2 on Wnt signalling in the presence of APP using reporter assays for Wnt-β-catenin and Wnt-PCP signalling. The ability of APP to potentiate Wnt-β-catenin signalling in response to Wnt3a was enhanced by the overexpression of LRP6, and attenuated by Vangl2 (Fig. 1g). Conversely, the ability of APP to potentiate Wnt-PCP signalling in response to Wnt5a was enhanced by Vangl2 and attenuated by LRP6 (Fig. 1h). These findings are all consistent with the effects of APP on Wnt signalling being mediated through interactions with either LRP6 or Vangl2.

### Swedish APP differentially affects Wnt-β-catenin and Wnt-PCP signalling

**Fig. 2:**
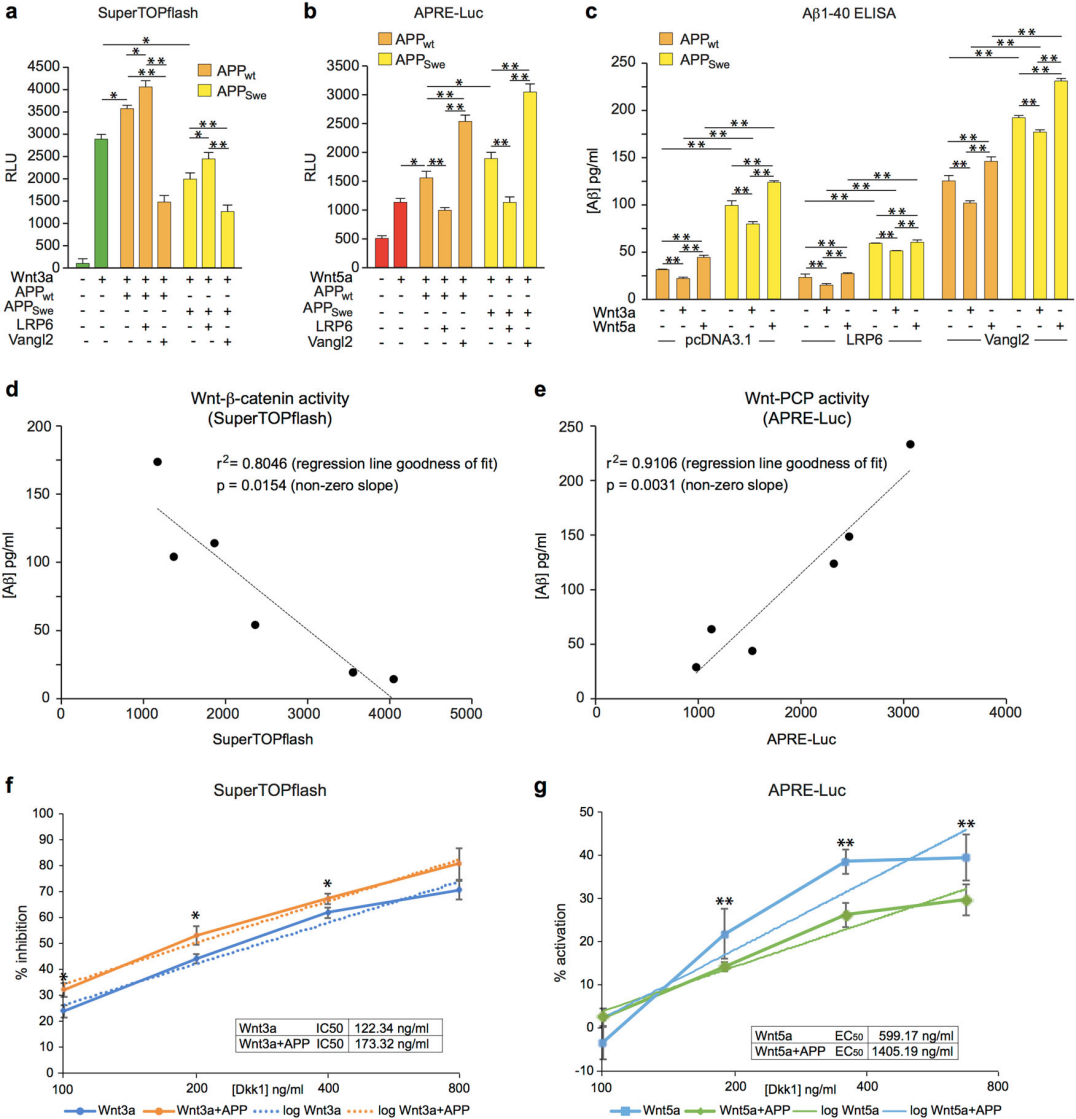
Familal and wild-type forms of APP differentially impact Wnt signalling. **a** Comparison of SuperTOPflash reporter activity in HEK293A cells expressing wild-type APP (APP_WT_) or the Swedish APP variant (APP_Swe_) indicates that the FAD form of APP is less able to promote Wnt-β-catenin signalling driven by Wnt3a, irrespective of co-receptor expression. **b** In contrast, APP_Swe_ enhances Wnt-PCP signalling driven by Wnt5a as measured by APRE activity, more so in the presence of the Wnt-PCP co-receptor Vangl2. **c** ELISA quantification of Aβ_1–40_ production in HEK293A cells demonstrates that Wnt-PCP components which promote PCP activity increased Aβ_1–40_ production. Conversely, promotion of Wnt-β-catenin activity reduced Aβ_1–40_ production. These data were obtained from the same cultures analysed in (**a**, **b**). **d** Regression analysis from paired data from **a** and **c** (Wnt3a-stimulated) demonstrates a strong negative correlation between the strength of Wnt-β-catenin signalling (SuperTOPflash activity) and ELISA measures of Aβ_1–40_ (*r*^2^ = 0.8046). **e** In contrast, regression analysis of the paired data from **b**, **c** (Wnt5a-stimulated) showed a strong positive correlation between Wnt-PCP activation (APRE-luc) and Aβ_1–40_ production (*r*^2^ = 0.9106). **f** Antagonism of Wnt-β-catenin signalling by Dkk1 was enhanced by the overexpression of APP in HEK293A cells as measured by SuperTOPflash reporter assays. **g** Dkk1 inhibition of Wnt-β-catenin signalling drove the activation of Wnt-PCP as measured by increased AP1 transcriptional activity. This effect of Dkk1 on Wnt-PCP signalling was enhanced by APP overexpression. Data are plotted as mean + /− standard deviation (*n* ≥ 3). Statistical significance was determined by one-way ANOVA and post-hoc Tukey’s test (*p < 0.05; **p < 0.01); *p* values are indicated for key comparisons only

An involvement of APP in Wnt signalling, which regulates synapse formation and stability, raises the question of whether familial AD mutations in APP may impact the activity of APP in Wnt signalling. Using the reporter gene assays in HEK293A cells, we compared the effects of the wild-type form of APP_695_ with the Swedish mutant form (KM670/671NL) (APP_Swe_). In contrast with wild-type APP which, as before, potentiated both canonical and non-canonical Wnt signalling, the Swedish mutant form of APP antagonised canonical Wnt signalling (Fig. 2a), and potentiated non-canonical Wnt signalling to a greater degree than wild-type APP (APP_WT_) (Fig. 2b). The pattern of LRP6 and Vangl2-modification of the Wnt signalling activities of APP_Swe_ was similar to that of APP_WT_, although APP_Swe_ still inhibited canonical signalling when co-expressed with LRP6 and enhanced non-canonical further still in the presence of exogenous Vangl2 (Fig. 2a, b).

### Aβ production is intimately linked with Wnt signalling

It is very well established that familial AD mutations of APP confer increased production of Aβ, and we have found that the Swedish variant shifts the balance of Wnt signalling activity away from Wnt-β-catenin towards Wnt-PCP. This suggests that there may be a direct link between the canonical and non-canonical Wnt pathways and the differential processing of APP. To investigate this, we assayed the amount of Aβ released into the media of HEK293A cells expressing APP_695_ in the absence of exogenous Wnt or in in the presence of Wnt3a or Wnt5a. As expected, cells expressing the Swedish mutant form of APP_695_ produced much more Aβ than the control wild-type-expressing cells. In cells expressing either wild-type or Swedish APP, the amount of Aβ produced was reduced in cells stimulated with Wnt3a, which promotes Wnt-β-catenin signalling, whereas Aβ production was enhanced in cells stimulated with Wnt5a, which promotes Wnt-PCP signalling (Fig. 2c). Co-expression of LRP6 with APP reduced the production of Aβ, while co-expression of Vangl2 with APP led to increased Aβ production (Fig. 2c). In each case Aβ production was higher under conditions that favour Wnt-PCP signalling and decreased under conditions that favour Wnt-β-catenin signalling. Comparison of signalling activity and Aβ production under each of the different conditions showed clearly that Aβ production was negatively correlated with Wnt-β-catenin signalling activity (Fig. 2d) and positively correlated with Wnt-PCP signalling (Fig. 2e).

### Functional interactions between APP and Dkk1

As the Wnt signalling modulator Dkk1 has been shown to be central in Aβ synaptotoxicity, we assessed the effects of APP on Dkk1-mediated modulation of canonical and non-canonical signalling activity. Overexpression of APP enhanced the inhibitory effects of Dkk1 on Wnt3a induced Wnt-β-catenin signalling, counteracting the enhanced activity resulting from APP overexpression and reducing the IC50 of Dkk1 to 122 ng/mL from 173 ng/mL in the absence of APP (Fig. 2f)

In contrast, the stimulatory effects of Dkk1 on Wnt-PCP signalling induced by Wnt5a were enhanced by APP overexpression, decreasing the EC50 of Dkk1 to 599 ng/mL from 1405 ng/mL (Fig. 2g). These data suggest that APP can enhance the potency of Dkk1 to modulate Wnt signalling.

### APP enhances synaptoxicity mediated by Dkk1

**Fig. 3:**
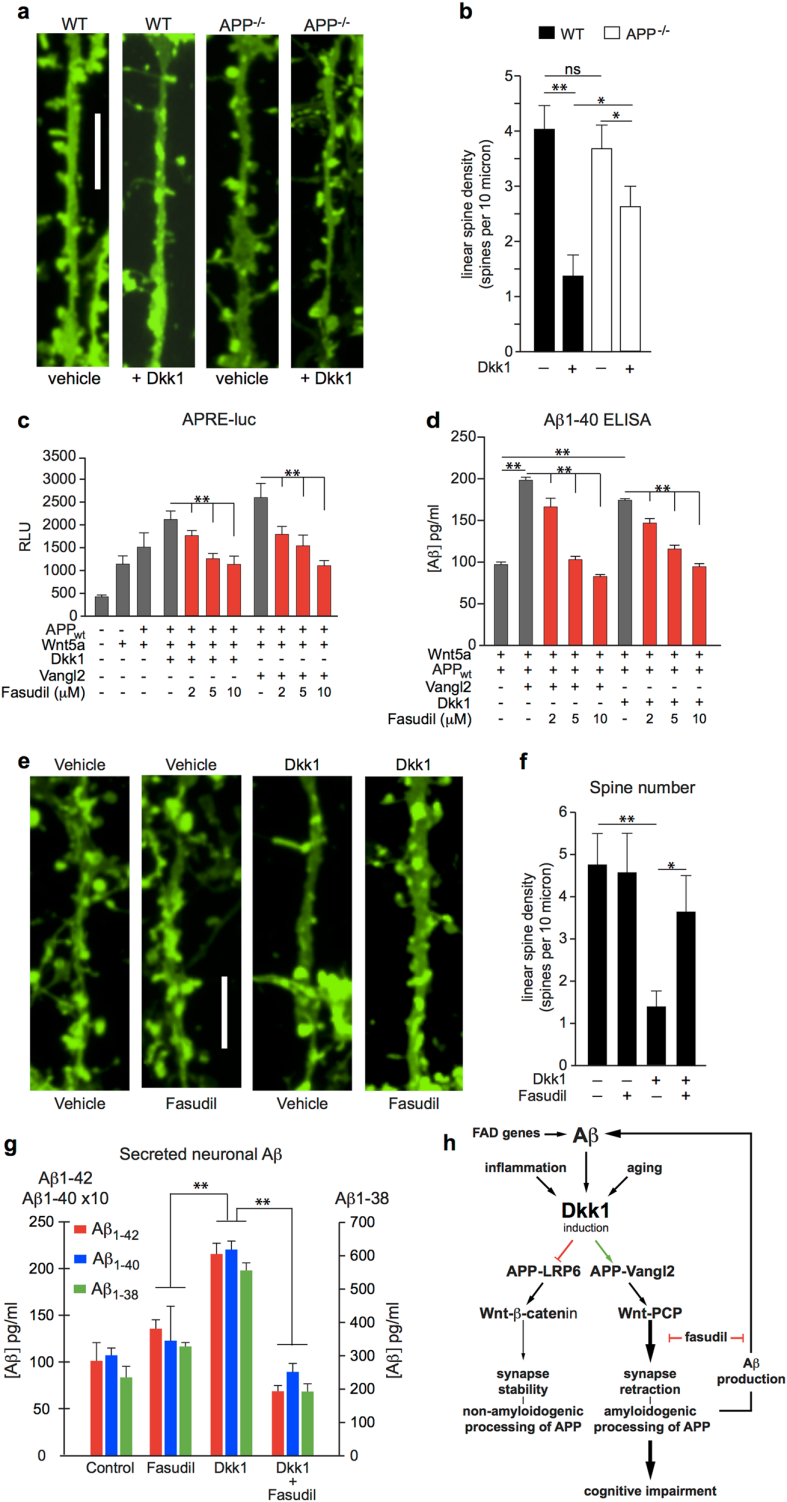
Dkk1-mediated synapse loss is APP-dependent and drives Aβ production. **a**, **b** Overnight treatment of primary cultures of wild-type mouse cortical neurons with Dkk1 (200 ng/mL) induced significant dendritic spine loss as visualised by DiO labelling. In APP^−/−^ neurons, dendritic spine density was similar to wild type, while Dkk1-mediated synapse loss was significantly reduced. **c** Fasudil exerted dose dependent inhibition of Wnt-PCP activity as measured by APRE reporter assay in HEK293A cells. **d** Inhibition of Wnt-PCP activation by fasudil also attenuated Aβ_1–40_ production in paired supernatants from (**c**). **e**, **f** Fasudil (10 µM) attenuated Dkk1 (200 ng/mL, 18 h) mediated dendritic spine loss in rat primary rat cortical neurons as visualised by DiO labelling and quantified by measures of dendritic spine density. **g** Aβ_1–38_, Aβ_1–40_, & Aβ_1–42_ levels in media collected from primary neurons in **f**. Dkk1 promoted synapse loss and also drove increased global Aβ production; the increased Aβ production was reversed by fasudil treatment. The three Aβ species measured responded in parallel to the different treatments. The data are plotted as mean + /− standard deviation (*n* = > 3). *P* values were determined by one-way ANOVA and post-hoc Tukey’s test (*p < 0.05; **p < 0.01); *p* values are indicated for key comparisons only. **h** Diagram showing the positive Aβ feedback loop whereby, through the induction of Dkk1, Aβ drives synapse loss which then drives further Aβ production. Blocking the Aβ-Dkk1-driven activation of Wnt-PCP with fasudil inhibits Aβ production

Aβ synaptoxicity is Dkk1-dependent^12,24^ and also appears to be APP-dependent^25^. The results described above show that APP enhances the effects of Dkk1, changing the balance of Wnt signalling away from the Wnt-β-catenin pathway and towards Wnt-PCP signalling. Given the known differential effects on synapses of Wnt-β-catenin and Wnt-PCP signalling, this suggests that APP might mediate the synaptotoxicity of Dkk1. To investigate this, the numbers of dendritic spines were counted in primary neuronal cultures from APP^+/+^ and APP^−/−^ mice following treatment with Dkk1 or vehicle (PBS) for 3 h. As previously reported, Dkk1-treatment markedly reduced the number of dendritic spines on APP^+/+^ neurons (Fig. 3a, b). In contrast, APP-deficiency protected neurons against the synaptotoxic activity of Dkk1 (Fig. 3a, b). These findings are consistent with the action of APP as a modulator of Wnt signalling described above.

### Synapse loss and Aβ production are intimately linked and amenable to pharmacological modulation

The Wnt-PCP signalling pathway acts through RhoA and ROCK^26^, and can be inhibited pharmacologically, for example, with fasudil^24^, a ROCK inhibitor that is in clinical use for the treatment of cerebral vasospasm. In reporter assays performed in HEK293A cells 10 µM fasudil completely reversed the stimulatory effect of Dkk1 and of Vangl2 on Wnt-PCP signalling (Fig. 3c). Fasudil also reversed the stimulatory effect of Vangl2 and Dkk1 on Aβ production in the same cells as determined by ELISA based quantification of Aβ_1–40_ levels in the paired culture media from the same experiments (Fig. 3d).

To investigate the relevance of the findings described above in a neuronal context, we examined the effects of fasudil on Dkk1-driven dendritic spine withdrawal and Aβ production in primary cultures of rat cortical neurons. Consistent with our previous findings^24^, treatment with Dkk1 resulted in substantial loss of dendritic spines, which was blocked by 10 µM fasudil treatment (Fig. 3e, f). The amounts of endogenous Aβ species (Aβ_1–38_, Aβ_1–40_ and Aβ_1–42_) secreted into the culture media by these treated neurons were determined using a multiplex assay (Fig. 3g). In addition to causing a significant reduction in the numbers of dendritic spines, Dkk1 treatment also resulted a substantial increase in levels of all three Aβ species (Fig. 3g). In the absence of exogenous Dkk1, fasudil had little effect on the amounts of Aβ secreted by the neurons. Notably, in parallel with the protective effect of fasudil on synapses (Fig. 3e, f), treatment with fasudil reversed the stimulatory effects of Dkk1 on Aβ production (Fig. 3g).

### Fasudil attenuates Aβ burden in vivo

**Fig. 4:**
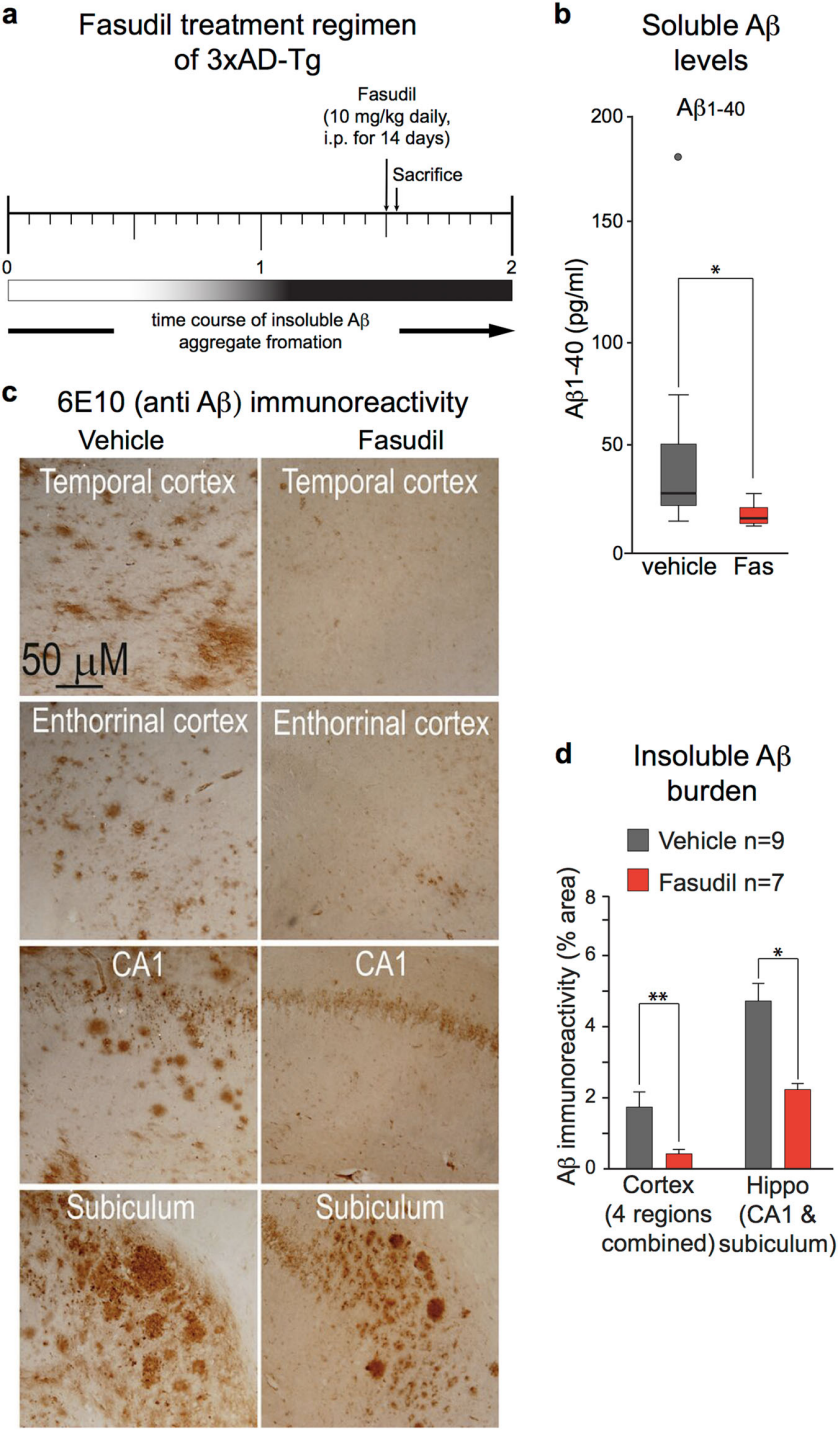
Fasudil reduced Aβ content and amyloid plaque load in 3xAD-Tg mice. **a** Schema of the treatment regime. Fasudil was administered daily to aged 3xAD-Tg mice for two weeks (10 mg/kg, by ip). **b** Protein extracts were generated from the frontal cortex and soluble Aβ_1–40_ levels determined by ELISA. Soluble Aβ_1–40_ levels were significantly reduced in fasudil treated animals (*p < 0.05, Mann–Whitney *U* test). **c**, **d** Brain sections from multiple regions were immunohistochemically labelled with anti-Aβ antibody 6E10. Fasudil treatment substantially and significantly reduced amyloid plaque burden in pooled cortical (frontal, temporal, parietal and entorhinal) and in pooled hippocampal regions (CA1 and subiculum). Data are plotted as mean + /− standard deviation. P values were determined by one-way ANOVA and post-hoc Tukey’s test (*p < 0.05; **p < 0.01)

We previously demonstrated that fasudil protects against both Aβ- and Dkk1-driven synapse loss and cognitive impairment^24^, and show above that fasudil can inhibit the production of Aβ by neurons stimulated with Dkk1. We therefore investigated whether fasudil might also attenuate amyloid pathology in vivo. These experiments used the 3xTG-AD transgenic mouse model^27^, which express human APP_Swe_, as well as mutant human tau (MAPT-P301L) and mutant presenilin-1 (PSEN1-M146V). In this model^27^, Aβ pathology is readily detectable at 12 months of age, and abundant in 18-month old mice, which show substantial soluble and insoluble amyloid load in brain, including plaque-like, Aβ-immunoreactive deposits (Fig. 4a).

Mice were treated at 18 months of age with 10 mg/kg fasudil or with vehicle (PBS), administered daily by i.p. injection, for 14 days. One day after the last injection, brains were collected, sagitally bisected, and fixed or snap frozen for subsequent histological and biochemical analyses. The rostral poles of snap frozen hemi-brains were homogenised and the levels of soluble Aβ_1–40_ measured by ELISA. Animals receiving fasudil had significantly lower soluble Aβ_1–40_ levels than controls (Fig. 4b). Fixed hemi-brains were sectioned and immunostained using the anti-APP/Aβ antibody, 6E10. IHC shows that administration of fasudil for just 14 days substantially reduced amyloid plaque burden, across all cortical brain regions examined, and in hippocampal regions CA1 and subiculum (Fig. 4c, d).

## Discussion

Loss of hippocampal synapses is pronounced in early Alzheimer’s disease and detectable in individuals with mild cognitive impairment^28,29^. The reduction in synapse number correlates with declining cognitive ability, thus a treatment that reduces or prevents pathological synapse loss would be expected to slow or halt cognitive decline. Indeed, protecting dendritic spines and maintaining structural plasticity has been shown to provide cognitive resilience^30^. Aβ is potently synaptotoxic, by understanding the mechanisms driving this property it may be possible to intervene to prevent synapse loss and preserve cognition, We have previously shown that Aβ-driven synapse loss is Dkk1-dependent and requires activation of the non-canonical Wnt-planar cell polarity pathway^24^. Here we extend this to show that Dkk1-driven synapse loss is also APP-dependent, with APP acting as a component of the Wnt-PCP pathway, co-activating Wnt-PCP through a direct protein-protein interaction with the Frizzled co-receptor protein Vangl2.

Dkk1 also modulates canonical Wnt-β-catenin signalling though, contrary to its effect on Wnt-PCP, it antagonises canonical Wnt signalling activity. Dkk1 does this by binding to and removing LRP6 from the canonical-Wnt co-receptor complex thereby preventing canonical Wnt activity^31,32^. Here we have shown that APP is also a component of the canonical Wnt signalling pathway, and that through a direct protein-protein interaction with the Frizzled co-receptor LRP6, in the absence of Dkk1, acts to promote canonical Wnt-βcatenin activity. It is now well established that synapse formation, stability and disassembly are Wnt regulated processes^16,18,19^. Activation of Wnt-β-catenin signalling stabilises synapses and promotes the formation of new ones, while Wnt-PCP signalling destabilises synapses and promotes the loss of synaptic connections. With APP acting as a co-activator of both Wnt signalling pathways and being expressed at synapses, APP can then be thought of as part a molecular switch mechanism, controlled by Dkk1, which regulates processes that determine if a particular synapse should be strengthened or removed. In this context, the properties of the Swedish familial AD form of APP we observed on Wnt signalling have particular interest. We found that the Swedish variant was a more potent co-activator of Wnt-PCP signalling than wild-type APP and, unlike wild-type APP, which co-activates Wnt β-catenin signalling, APP_Swe_ suppressed this branch of Wnt. These effects of Swedish APP compared to wild-type APP would each act in the direction that would reduce synapse stability and favour synapse disassembly. This then suggests that altered co-activation of Wnt signalling by APP_Swe_ could contribute to synapse loss by both reducing synaptogenesis and enhancing synapse withdrawal, at least in those familial cases of AD carrying the Swedish mutant form of APP. If this extends to other FAD forms of APP it is of importance for our understanding of the early-onset form of AD due to mutations in APP.

Amyloidogenic processing of APP occurs in endosomes, following clathrin-mediated endocytosis^6,7^, and endosomal trafficking of FAD mutant forms of APP, including APP_Swe_, has been shown to differ from that of wild-type APP^33^. Wnt-PCP signalling is also tightly linked to clathrin-mediated endocytosis^34^. This suggests that the altered trafficking of APP_Swe_, which results in enhanced Aβ production may also be responsible for the enhanced co-activation of Wnt-PCP signalling by this variant. Consistent with this is the data we present here that Aβ production correlates positively with Wnt-PCP signalling activity and inversely with Wnt-β-catenin signalling. We would propose then that the signalling activity of APP and the generation of Aβ are likely interrelated processes. Under conditions that favour synapse stabilisation, i.e., when APP co-activates canonical Wnt, APP is non-amyloidogenically processed and Aβ production is reduced, conversely under conditions favouring retraction, when APP is acting to promote Wnt-PCP, it is more likely to be amyloidogenically processed and Aβ production is increased.

Given our data indicate that canonical Wnt favours α-secretase cleavage, and non-canonical Wnt activity favours β-secretase cleavage of APP. It is of interest that Parr et al.^35^ have shown that the expression of BACE1, the major β-secretase is negatively regulated by canonical Wnt activity and Wan et al.^36^, that the expression of ADAM10, the major α-secretase, is positively regulated by canonical Wnt signalling. These observations indicate that the expression of the major APP secretases are regulated in a manner that is in keeping with our data and indicates that such process would act to compliment each other.

We^10,24^, and others^11,12^ have previously shown that multiple aspects of Aβ toxicity are mediated by its ability to induce Dkk1 expression. While Dkk1 has many important developmental functions^37^, it is barely expressed in the brain of young adults. Notably, Dkk1 expression has been found to increase with age, and the knockout of Dkk1 in adult mouse brain has been found to counteract normal age-related cognitive decline through restoration of neurogenesis and increased dendritic complexity^38^. These properties make Dkk1 an attractive target for therapeutic intervention. In addition to many neurotoxic properties of Dkk1, we have shown that Dkk1 potently stimulates the neuronal production of Aβ. Given that Aβ drives the neuronal expression of Dkk1^10–12^, this demonstrates the existence of a positive feedback loop, which we present schematically in Fig. 3h. In this model, Aβ synaptotoxicity drives further Aβ production through a process which is dependent on both Dkk1 and APP and the co-receptor proteins LRP6 and Vangl2. While other mechanisms of Aβ toxicity, over and above those due to effects on Wnt signalling, most certainly exist, we would suggest that blocking this feedback loop offers a potential mechanism through Alzheimer’s disease neuropathology could be effectively modified.

The Dkk1-mediated toxic effects of Aβ are depend on activation of the PCP branch of Wnt signalling^10,24^. Wnt-PCP itself has two arms, signalling through c-Jun N-terminal kinase (JNK1) and c-Jun, or through RhoA and Rho-associated kinase (ROCK). Aβ neurocytotoxicity is dependent on the JNK/c-Jun arm of the Wnt-PCP pathway and requires gene transcription^10^, while Aβ synaptotoxicity is mediated predominantly through Dkk1 and the Rho/ROCK arm of the Wnt-PCP pathway^24^. We previously demonstrated that inhibition of Wnt-PCP signalling using fasudil, a ROCK inhibitor, protects against both Aβ- and Dkk1-driven synapse loss in vitro, and in vivo against acute Aβ-induced cognitive impairment^24^. Similarly, it has been reported that fasudil protected against dendritic atrophy in an AD mouse model^39^, and against hippocampal neuronal death induced by intracranial injection of Aβ^40^. Others have shown that, in young AD model mice, Aβ production can be reduced by inhibition of Rho^41^ or ROCK^42^. And in support of assessing fasudil further as a potential treatment for AD not only does the drug to reduce Aβ production from neuronally expressed human APP^42^, supporting of our own data here, but it has also been shown to ameliorate tau pathology, albeit in models of other forms of dementia, that like AD, include pathological tau aggregates^43^. That fasudil is a pan ROCK inhibitor, with little difference in selectivity towards either ROCK1 or ROCK2, may not be problematic given both forms of ROCK have been shown to have similar effects on APP processing^42,44^.

These data are consistent with our findings that fasudil treatment greatly reduced both soluble and insoluble Aβ burden. Even in mouse models engineered to deposit amyloid aggressively, accumulation of Aβ is slow. At the time of treatment, the mice in our experiments had very substantial amyloid deposits that had progressively accumulated over 18 months. Against this timescale, the magnitude of the response to treatment is consistent with rapid turnover of Aβ which, in the disease state, is counterbalanced by accelerating production of Aβ driven by positive feedback.

It is over 30 years since the APP gene was molecularly cloned and the protein it encoded for found to have characteristics of a glycosylated cell surface receptor^1^. Recently, there has been a resurgence of interest in the role of APP as a receptor and as a cell adhesion protein (reviewed in^8^ and^45–47^, respectively). The data we present here indicate that APP, if not a true receptor protein *per se*, can act as a component of both the Wnt-β-catenin and Wnt-PCP co-receptor protein complexes. As Wnt-β-catenin and Wnt-PCP are regarded, respectively, as promoting cell adhesion and cell separation this may underpin many of the reports suggesting APP acts as a neuronal adhesion molecule and may, in part at least, account for the apparently contradictory reports concerning APP and synapse stability, given an ability to act in both arms of Wnt signalling.

Due to the recent series of failures of therapeutics targeting Aβ in clinical trials, the amyloid cascade hypothesis has come under intense criticism. The existence of a positive feedback loop driving Aβ production may provide a partial explanation for clinical trial failures because of residual Aβ leading to re-accumulation of Aβ. Indeed, it is predicted that there will be a rebound effect unless normal or near normal Aβ concentrations are achieved. While our data largely support of the amyloid cascade hypothesis, the findings reported here indicate that a central component of Alzheimer’s disease pathology is the physiological function of APP rather than Aβ production *per se*. Further, it highlights the downstream target of Aβ and modulator of the APP-Wnt activity, Dkk1, as key element, and a possibly more amenable therapeutic target for combatting AD than Aβ has thus far proven to be.

## Methods

### Genetically modified mice

**APP-KO mice** (B6.129S7-Ap ptm1Dbo/J) were obtained from the Jackson Laboratory and maintained on the C57Bl/6 background. Natural matings (APP KO x APP KO or C57BL/6 x C57BL/6 to provide KO and wild-type material respectively) were set-up, and mating plugs were checked. On E16.5 the brains from APP KO or C57BL/6 wild-type embryos were collected. These were dissected out on ice-cold PBS (-ve calcium) and the cortex and hippocampus collected to provide neurons for culture described below.

**3xTG-AD mice** were generated by the La Ferla group^27^ and were obtained from Jackson Laboratory Animals on a C57BL6/129 genetic background. The line harbor three mutant human transgenes, APP (KM670/671NL), MAPT (P301L) and PSEN1 (M146V). All mice were housed and treated in compliance with UK Home Office legislation under the Animals (Scientific Procedures) Act, 1986.

### Preparation of rodent cortical neurons

Neurons were prepared from E18 embryonic Sprague Dawley rat or E16.5 mouse cortices as described previously (Sellers et al., 2017). Briefly, cells were seeded on 16 mm glass coverslips (VWR) coated with poly-D-lysine (0.2 mg/ml, Sigma). Cells were cultured in neurobasal supplemented with B27 supplement, 0.2 mM glutamine and 1% penicillin/streptomycin (Thermo Fisher). Cultures were maintained at least 21 days in vitro (DIV) before use.

### Pharmacological treatments of neuronal cultures

All pharmacological treatments were performed in artificial cerebral spinal fluid (aCSF): 125 mM NaCl, 2.5 mM KCl, 26.2 mM NaHCO_3_, 1 mM NaH_2_PO_4_, 11 mM glucose, 5 mM Hepes, 2.5 mM CaCl_2_, 1.25 mM MgCl_2_, and 0.2 mM APV. Neuronal cultures were pre-treated with inhibitor compounds for 30 min prior to application of Dkk1 recombinant protein (R&D systems). Fasudil (SelleckChem) were dissolved in water and used at concentrations indicated in the text.

### DiO labelling, imaging and quantification of dendritic spines

After treatment neurons were fixed in 2% paraformaldehyde and fluorescently labelled using Vybrant™ DiO Cell-Labeling Solution (Life technologies), as described previously^48^ and mounted onto glass slides using mowiol mounting media.

Images were acquired with a Leica SP-5 confocal microscope using a ×63 oil-immersion objective (Leica, N.A. 1.4) as z-series, with an optical slice thickness of 0.5 µm, imaging parameters remained constant throughout. To identify hidden dendritic spines, the resulting pictures were then reconstructed as a 2D maximum projection. Spine numbers was quantified using imageJ from eight neurons selected at random (per condition, performed in duplicate), and expressed as spine density/ 100 µm length. Analysis was performed blind to condition. Cultures undergoing direct comparison were stained simultaneously and imaged with the same acquisition parameters. Data are expressed as spine density/10 µM, error bars represent standard deviation from a minimum of 3 independent experiments (10 neurons/ experiment selected at random).

### Luciferase Reporter assays

SuperTOPflash and APRE-luc (pGL4.44[luc2P/AP1 RE/Hygro], Promega), reporter gene assays, conducted in cell lines, were performed as previously described^49^. All other constructs used in this study including Wnt3a, Wnt5a, Vangl2 and LRP6 were expressed in versions of pcDNA 3.1 (Invitrogen). Briefly, HEK293A cells were transfected with Fugene 6 (Promega) according to manufacturer’s instructions. Cells were incubated for a minimum incubation of 18 h to allow for sufficient expression and read using One-Glo or Dual Glo reagents (Promega, UK). All reporter assay data are plotted as mean + /− standard deviation (*n* ≥ 3). Statistical significance was determined by one-way ANOVA and post-hoc Tukey’s test (*p < 0.05; **p < 0.01); *p* values are indicated for key comparisons only.

### Aβ quantification

Conditioned medium for analysis was spun at 800 × *g* to remove cellular debris, and supernatant stored at −20 °C until use. Human amyloid-β(1–40) production in cell lines was measured using a quantikine ELISA kit (R&D systems). Amyloid-β production (1–38, 1–40, 1–42) in primary rat neurons was measured using a multiplexed immunoassay (Meso Scale Diagnostics). All assays were performed following the manufacturer’s instructions. Data are plotted as mean+/− standard deviation (*n* ≥ 3). Statistical significance was determined by one-way ANOVA and post-hoc Tukey’s test (*p < 0.05; **p < 0.01); *p* values are indicated for key comparisons only.

### Peptide array mapping

Peptide libraries spanning the full length of APP were produced by automatic SPOT synthesis and synthesised on continuous cellulose membrane supports on Whatman 50 cellulose membranes using Fmoc-chemistry with the AutoSpot-Robot ASS 222 (Intavis Bioanalytical Instruments AG, Köln, Germany) as we have previously described^50^. Arrays were incubated with recombinant human LRP6 (Fc-chimera, R&D systems) or total lysate from HEK293A cells overexpressing HA-tagged Vangl2. Binding was visualised using either an anti-HA tag (Abcam, ab9110) antibody or anti-Human IgG Fc Cross-Adsorbed Secondary Antibody, DyLight 680 (Thermo Fisher, SA5-10138) and imaged on a Chemidoc system with Image Lab software (BioRad).

### Immunoprecipitation

Lysates were extracted from rat neurons (21 d.i.c.) in lysis buffer [25 mM Hepes, 2.5 mM EDTA, 50 mM NaC1, 50 mM NaF, 30 mM sodium pyrophosphate, 10% (v/v) glycerol, 1% (v/v) Triton X-100, pH 7.5, containing Complete™ EDTA-free protease inhibitor cocktail tablets (Roche)] as previously described^50^ with 10% of total lysate being retained as input. Immunoprecipitation was performed using SureBeads™ Protein G Magnetic Beads (BioRad) with either an anti-LRP6 antibody (Abcam, ab75358) or anti-Vangl2 antibody (Santa Cruz, sc-46561) following manufacturers guidelines. Samples were resolved by SDS-PAGE followed by western blotting. APP was visualised using an anti-APP rabbit antisera 369 (a kind gift from Professor Joseph Buxbaum)^51^, using the appropriate fluorescently conjugated secondary antibody and imaged on a Chemidoc system with Image Lab software (BioRad).

### Amyloid burden in vivo

#### Animals

Triple Transgenic mice carrying Swedish FAD APP_695_, PSEN M146V and MAPT P301L were obtained from The Jackson Laboratory, bred and aged to 18 months, *N* = 17 (9 female, 8 male). Fasudil HCL (10 mg/kg) or an equivalent vol of vehicle (0.9% saline) was administered via intraperitoneal injection once a day for 14 consecutive days. On the final day, the animals received a further injection (number 15) and then were sacrificed 1 h later by cervical dislocation, brains rapidly removed, hemisected, half being flash frozen on liquid 2-methylbutane at −55 °C for biochemical analysis and the remaining half post-fixed in 4% PFA for immunohistochemical staining. *Tissue samples*. Rostral pole of frozen frontal lobe were cut weighed and homogenised on ice in a 8 x volume of PBS to which protease inhibitors (Roche), phosphatase inhibitors (Phos-STOP), NaF (10 mM), and β-glycerol phosphate (2 mM) had been added. Of the *N* = 17 mice 10 (4 male, 6 female) received vehicle and 7 (4 male, 3 female) fasudil. The homogenates were subjected to centrifugation at 100,000 g at 4 °C for 20 min. The supernatant (S1) and pellet (P1) were collected and stored with at −80 °C.

### Plaque labelling and Aβ densitometry

APP immunohistochemistry, 30 µm-thick sagittal sections from fixed brains were immunostained as previously described^52,53^, using the anti-Aβ antibody, 6E10 (Covance Cat: 803001). Animals were perfused through the left ventricle with saline solution, followed by 4% paraformaldehyde in 0.1 M phosphate buffer, pH 7.4, for 15 min. Brains were removed and cryoprotected by soaking in 30% sucrose solution in phosphate buffer until they sank. Parallel series of 30 µm thick coronal sections were obtained in a freezing microtome. Sections from vehicle and fasudil treated animals were processed with the same solutions and processing times. Sections were rinsed in 100 mM Tris-HCl, pH 7.6, and 225 mM NaCl (TBS). Antigen retrieval for APP/Aβ detection was performed by incubating the sections for 5 min in formic acid. Tissue peroxidase was inactivated by incubating in 10% methanol and 10% hydrogen peroxide in TBS for 30 min. After three washes in TBS, sections were incubated for 2 h in blocking solution (10% normal goat serum, 0.3% Triton X-100 in TBS), and then for 16 h at 4 °C with mouse anti-APP/Aβ antibody, clone 6E10 at a 1/500 dilution (Covance Cat: 803001). Sections were rinsed in TBS, then incubated with biotinylated goat anti-mouse IgG at 1/1000 dilution for 1 h, and then incubated with the Vectastain ABC HRP kit (Vector laboratories) following manufacturer’s instructions. Sections were subsequently developed in 0.05% 3′−3′ diaminobenzidine tetrahydrochloride (Sigma-Aldrich) in Tris-HCl buffer, pH 8.0 containing 0.003% hydrogen peroxide (Sigma-Aldrich) for 5 min. Immunoreagents were diluted in 1% goat serum and 0.2% Triton X-100 in TBS. Sections were mounted on gelatin-coated slides, air-dried, and finally dehydrated in graded alcohols, cleared in xylene and coverslipped. Control sections were treated with the same protocol but omitting the primary antibody. A mean of 25 images from each brain were acquired using the same exposure settings. The captured images were normalised into 8-bits grey scale employing Adobe Photoshop CS4 v11.0 and opened in ImageJ v1.48 software. The threshold being set for each image using the histogram mean at the same standard deviation. To eliminate background the particle size pixel was set at 20-infinity pixel. Plaque-like deposits were quantified in frontal, temporal, parietal and entorhinal cortex (grouped as cortex) and in CA1 and subiculum (grouped as hippocampus) employing the “analyze particles” plug-in for Image J v1.48 software. Amyloid burden was calculated as the area occupied by the immunoreactive plaque-like structures expressed as % of area. Mean values are mean ± SEM (Vehicle, *n* = 9 and Fasudil, *n* = 7). Statistical analysis was performed with Student’s *t* test comparing Fasudil vs. vehicle treated mice. Analysis was performed blind to condition

### Statistical analysis

Statistical analysis was performed using IBM SPSS v22 as indicated in the text. Comparisons from two samples were performed using students *T* test and multiple comparisons effects were probed by 1- or 2-way ANOVAs with a post-hoc Tukey correction. Error bars represent standard deviation, where n represents independent experiments.

## Electronic supplementary material

Supplemental Figure 1
