# Supplementary figures and images for: A role for APP in Wnt signalling links synapse loss with β-amyloid production
Source: Transl Psychiatry. 2018 Sep 20;8:179. doi: 10.1038/s41398-018-0231-6 (PMC6145937; doi:10.1038/s41398-018-0231-6)

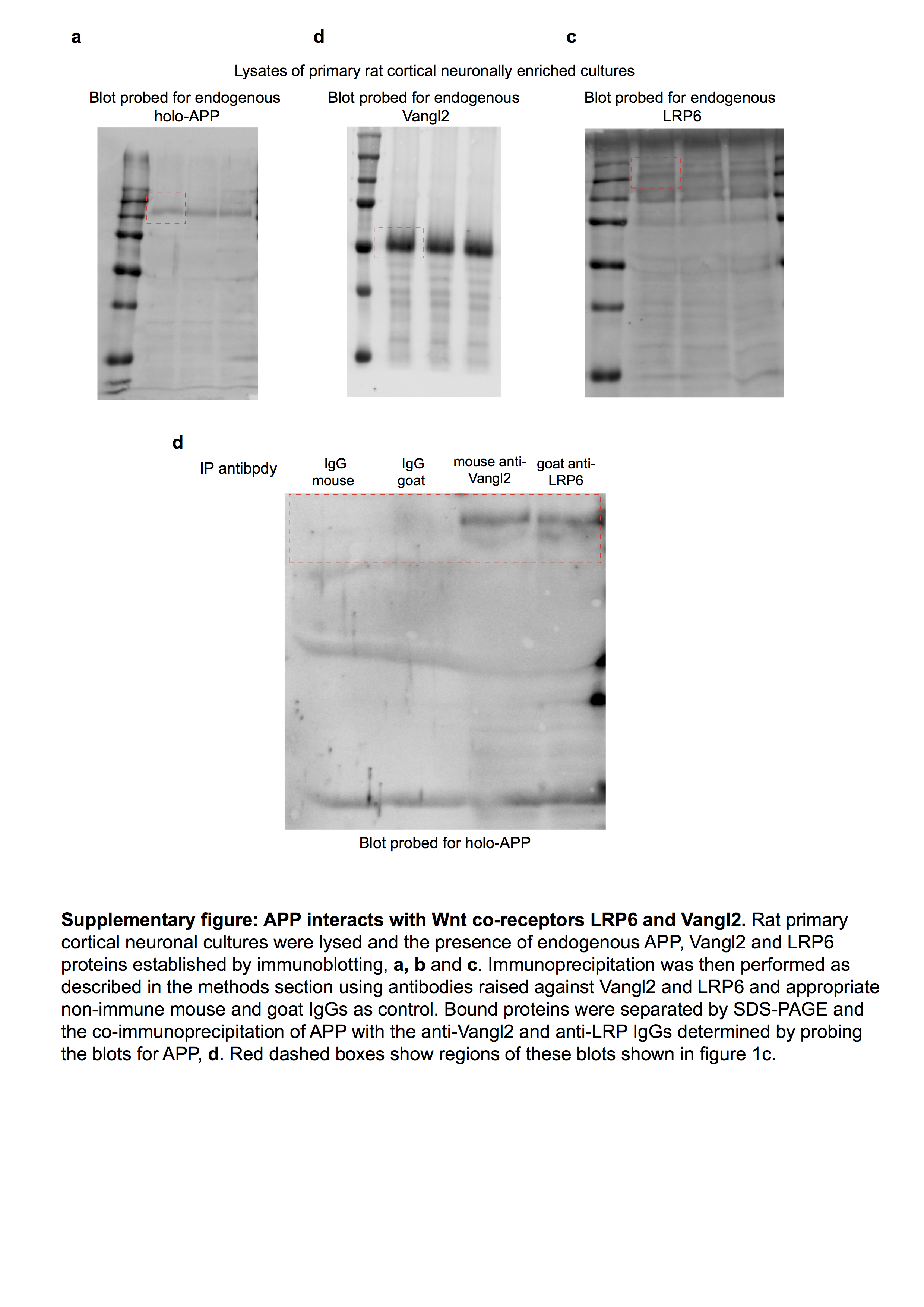

Supplement: Supplementary file 1 — Supplemental Figure 1 [file 41398_2018_231_MOESM1_ESM.tif]
